# Efficacy of chemotherapeutics on classic and non-classic Kaposi sarcoma: a single-center retrospective real-world study

**DOI:** 10.17305/bjbms.2020.5329

**Published:** 2021-12

**Authors:** Sibel Oyucu Orhan, Ahmet Bilgehan Şahin, Erdem Çubukçu, Adem Deligönül, Birol Ocak, Bedrettin Orhan, Türkkan Evrensel

**Affiliations:** 1 Department of Medical Oncology, Faculty of Medicine, Bursa Uludag University, Bursa, Turkey; 2 Division of Hematology, Department of Internal Medicine, Faculty of Medicine, Bursa Uludag University, Bursa, Turkey

**Keywords:** Kaposi sarcoma, chemotherapy, pegylated liposomal doxorubicin, paclitaxel

## Abstract

Kaposi sarcoma (KS) is a rare disease, and especially for classic KS, a gap exists in the literature about which chemotherapeutics should be given. Here we present our institutional data on the demographic characteristics, treatment, and treatment efficacy in 16 patients with KS treated with chemotherapy. We retrospectively analyzed the demographic and clinical characteristics of and the chemotherapeutic agents administered to the 16 patients with KS diagnosed in our center based on the medical records obtained. The median age, gender, KS type, involved site, cytotoxic agents administered, progression-free survival (PFS), overall survival (OS), objective response rate (ORR), disease control rate (DCR), and safety profiles of the patients were evaluated. The median age at disease onset was 61.07 years (range, 39.4–85.8 years). Among the patients, one had immunosuppression-related KS, four had acquired immune deficiency syndrome-related KS, and 11 had classic KS. Regarding the first-line cytotoxic therapy, seven patients received pegylated liposomal doxorubicin (PLD), six received paclitaxel, two received oral etoposide, and one received the doxorubicin, bleomycin, and vincristine regimen. The Kaplan–Meier analysis showed that the PFS was 39.9 months (95% confidence interval (CI), 7.7–72.0). In the first-line setting, a significant difference in PFS was observed between the PLD- and paclitaxel-treated groups (unreached vs. 12.8 months; p = 0.033). The OS was 66.1 months (95% CI, 30.2–102.0). The ORR and DCR of the 16 patients were 43.8%, and 81.3%, respectively. No grade 3 or 4 toxicity was observed. This retrospective study showed that among the most preferred chemotherapeutic agents, PLD seems better than paclitaxel in terms of PFS and response rates, and it showed a good safety profile in patients with KS.

## INTRODUCTION

Kaposi sarcoma (KS) is a chronic, multifocal, angioproliferative disease arising from the vascular endothelium and has a distinct clinical course [1,2]. This disease was named after Moritz Kaposi, who named the disease himself in 1872 and defined it as the “idiopathic multiple pigmented sarcomas of the skin” [3]. Four clinical subtypes of KS have been described: classic KS (CKS), KS due to immunosuppression, acquired immune deficiency syndrome (AIDS)-related (epidemic) KS, and endemic African KS [4]. CKS is mostly seen in elderly Eastern European Jewish or Mediterranean men with a male-to-female ratio of 5:1 [5,6].

KS is a chronic disease persisting for many years, but it does not usually lead to death [7,8]. The lesions present as purplish to reddish-blue well-demarcated macules, plaques, and nodules and usually start at the distal extremities, particularly in the lower legs and feet [5]. Lymphedema, edema, pain, ulceration, bleeding, and functional impairment may accompany these skin lesions [9]. In advanced cases, involvement of lymph nodes and visceral organs is observed.

KS treatment aims to decrease the number and size of lesions and to ease the disease symptoms, such as bleeding, pain, lymphedema, and edema; furthermore, it aims to delay disease progression. In advanced cases, pegylated liposomal doxorubicin (PLD) and paclitaxel are the recommended first-line systemic treatment independent of the subtype [6]. Other treatment options for KS include vinblastine, bleomycin, etoposide, vinorelbine, gemcitabine, and interferon alfa 2a and 2b [6,10,11]. Most published studies on AIDS-related KS are prospective or retrospective in nature. In addition, prospective studies on CKS are rare, and the treatment experiences were gained mostly through retrospective trials.

Thus, this study presents our institutional data on the demographic characteristics, treatment, and efficacy of treatment in 16 patients with KS treated with chemotherapy.

## MATERIALS AND METHODS

The demographic and clinical characteristics of and the chemotherapeutic agents administered to the 16 patients diagnosed with histopathologically confirmed KS who received chemotherapy at the Bursa Uludag University Hospital between January 2008 and June 2020 were retrieved from the medical files and retrospectively analyzed. The median age, gender, Eastern Cooperative Oncology Group (ECOG) performance status score, KS type, site of involvement, cytotoxic agents administered, PFS, OS, ORR, DCR and safety profiles of these patients were evaluated.

### Ethical statement

This study was conducted according to the institutional research committee’s ethical standards and the 1964 Declaration of Helsinki. The clinical research ethics committee of the Bursa Uludag University Faculty of Medicine approved this study (Decision No: 2020-6/23).

### Statistical analysis

Continuous variables were expressed as median (range) values, and categorical variables were expressed as frequency along with their corresponding percentage values. The patients’ response was evaluated according to the Response Evaluation Criteria in Solid Tumors. Patients with cutaneous involvement only were evaluated based on the AIDS Clinical Trials Group response criteria. PFS was calculated from the beginning of chemotherapy treatment to disease progression or death from any cause. The OS was determined from the time of diagnosis until death from any cause. The ORR was defined as the proportion of patients who achieved complete response (CR) or partial response (PR). In addition, the DCR was defined as the percentage of patients who had CR, PR, and stable disease (SD). Toxicity was assessed using the Common Terminology Criteria for Adverse Events version 5.0. Survival analysis was performed using the Kaplan–Meier method. Data were analyzed using Statistical Package for the Social Sciences, version 23, and statistical significance was set at the 5% type-I error level.

## RESULTS

Sixteen patients with KS were enrolled in this study, and their demographic data and treatments are shown in Table 1. The median age at disease onset was 61.07 years (range, 39.4–85.8 years). Fourteen (87.5%) patients were male and two (12.5%) were female. Among all patients enrolled in this study, one had immunosuppression-related KS, four had AIDS-related KS, and 11 had CKS. The patient with immunosuppression-related KS had membranous glomerulonephritis and received steroids, cyclophosphamide, cyclosporin, and azathioprine. Moreover, among the 16 patients, five (31.25%) had cutaneous involvement only, one (6.25%) had lymph node involvement only, and 10 (62.5%) had multiple-site (cutaneous, mucosal, lymph node, and visceral) involvement. The indications for chemotherapy are shown in Table 2. In total, 56% of the patients received chemotherapy due to symptomatic visceral, lymph node, or bone involvement. Regarding the first-line cytotoxic therapy, seven patients received PLD, six received paclitaxel, two received oral etoposide, and one received the doxorubicin, bleomycin, and vincristine (DBV) regimen. Six patients received second-line chemotherapy, and three received third-line chemotherapy. The clinical course for each patient is shown in Table 2. The median follow-up period was 44.2 months (range, 2–139 months). The median number of chemotherapy cycles administered as the first-line treatment was 6 (range, 3–13).

**TABLE 1:**
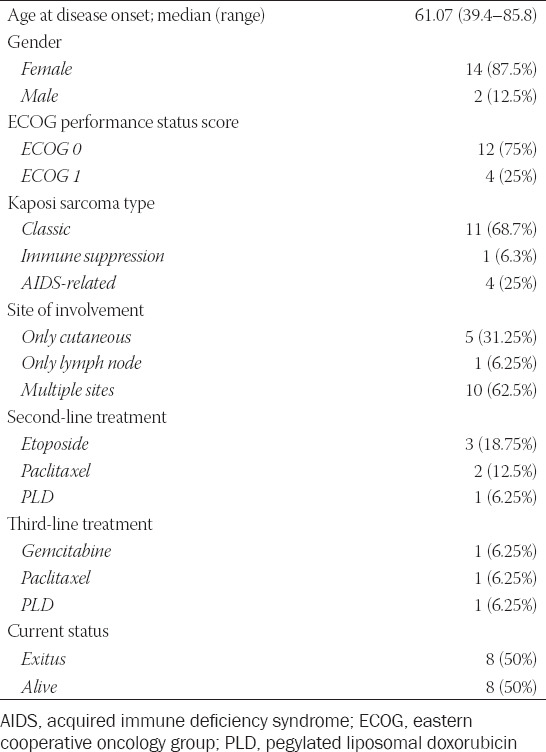
Baseline patient and disease characteristics

**TABLE 2:**
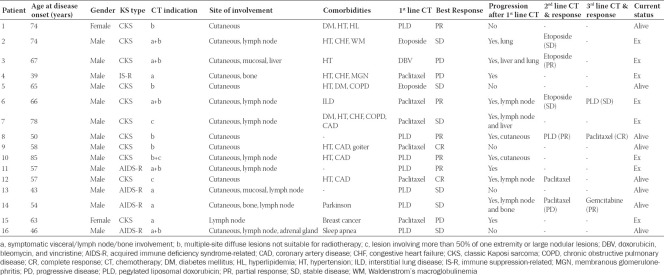
The patients’ clinical course.

**Figure 1:**
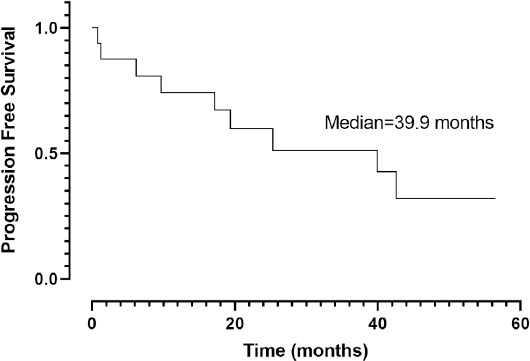
Kaplan Meier curve of progression-free survival of patients

**Figure 2:**
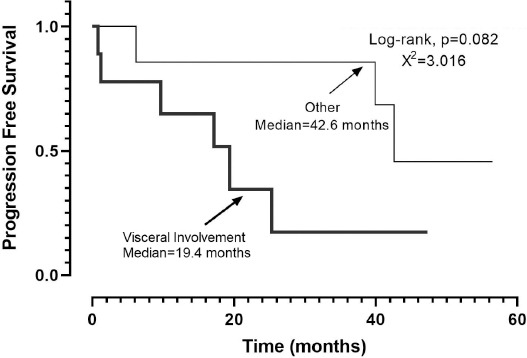
Progression-free Survival; Visceral involvement vs Other

**Figure 3:**
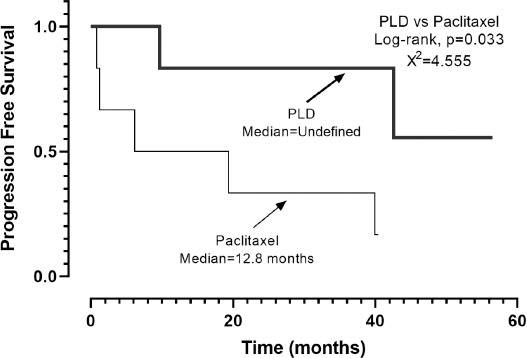
Progression-free Survival; Pegylated Liposomal Doxorubicin (PLD) vs Paclitaxel

**Figure 4:**
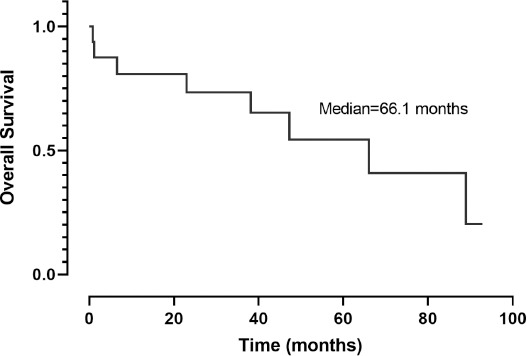
Kaplan Meier curve of overall survival of patients

The Kaplan–Meier analysis revealed that PFS was 39.9 months (95% CI, 7.7–72.0) (Figure 1). When the chemotherapy indication was symptomatic visceral involvement, along with lymph node and bone involvement, PFS was 19.4 months (95% CI, 7.9–30.9; *p* = 0.082) (Figure 2). Symptomatic visceral, lymph node, or bone involvement was present in three of the six patients who received paclitaxel and four of the seven patients who received PLD. No statistical significance in terms of visceral, lymph node, or bone involvement was observed between paclitaxel- and PLD-treated patients (*p* = 0.797). In the first-line setting, a significant difference in terms of PFS was observed between the PLD- and paclitaxel-treated groups (unreached vs. 12.8 months; *p* = 0.033) (Figure 3). The OS was 66.1 months (95% CI, 30.2–102.0) (Figure 4), and the OS rate was 80.8% in the first year, 73.4% in the third year, and 54.4% in the fifth year.

The ORR and DCR of the 16 patients were 43.8% and 81.3%, respectively. Among all patients, two (12.5%) achieved CR, five (31.3%) achieved PR, six (37.5%) had SD, and three (18.7%) had progressive disease (PD); the efficacy outcomes are shown in Table 3. The ORR and DCR for PLD and paclitaxel were 57.1% and 50%, respectively, and 100% vs 66.7%, respectively.

**TABLE 3:**
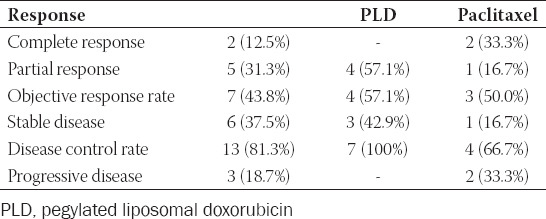
Response to treatment (n (%)).

In the first-line setting, adverse events were observed in five patients. Grade 2 peripheral sensory neuropathy was observed in only one of the six patients who received paclitaxel. Details on adverse events are shown in Table 4.

**TABLE 4:**
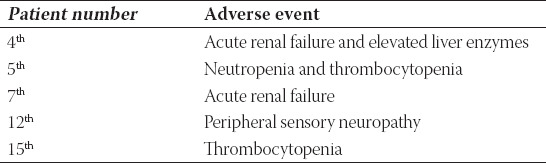
Adverse events

## DISCUSSION

KS is a rare disease and is mostly seen in the Mediterranean region. It is characterized by a slow progression during its course, and patients rarely need systemic treatment. The KS subtype, age, and comorbidities are essential parameters in determining the systemic treatment to be administered. However, no cytotoxic chemotherapeutic agent has been approved for specifically treating CKS, whereas several drugs against AIDS-related KS have been approved. Moreover, most published studies on AIDS-related KS are prospective or ­retrospective in nature, whereas prospective studies on CKS are rare, and treatment experiences were gained mostly through retrospective trials and case series. Only one randomized trial was conducted in which two systemic therapies were compared in the literature [12]. In that trial, 65 patients with CKS were randomly assigned to two groups: the oral etoposide and intravenous vinblastine groups. No significant differences in the response rate (74% vs. 58%, respectively) or survival was found between the two treatments. We believe that single-center retrospective studies are crucial in determining the best therapeutic option, especially for patients with CKS.

This study presents our institutional data on various systemic chemotherapeutic agents used in patients with KS and compares PLD and paclitaxel in terms of response rates and PFS when used in a first-line setting. To our knowledge, this retrospective study is the only study comparing PLD and paclitaxel used in patients with CKS and AIDS-related KS. The results of this study showed that PFS was significantly longer in patients treated with PLD than that in patients who received paclitaxel, and no PLD-related toxicity was observed.

PLD and paclitaxel were both used as active cytotoxic agents to treat advanced symptomatic KS. Because KS treatment is considered palliative rather than curative and each agent has a different degree of toxicity, we compared the effectiveness and toxicity of these two agents as first-line therapies for patients with advanced KS. A randomized controlled study involving 73 patients with AIDS-related KS, which is thus far the only trial comparing PLD and paclitaxel, has reported comparable response rates (56% vs. 46%, respectively; *p* = 0.49), median PFS (17.5 months vs. 12.2 months; *p* = 0.66), and 2-year survival rates (79% vs. 78%; *p* = 0.75) for PLD and paclitaxel, although a higher toxicity of approximately grade 3–5 was observed in paclitaxel (84% vs 66%; *p* = 0.077) [13]. In this study, the OS was 80.8% for the first year and 73.4% for the third year. Most patients (13 of 16) received PLD or paclitaxel in the first-line setting, contributing to the obtained OS results, which were similar to those reported in the aforementioned study. In terms of PFS, the median PFS for PLD was not reached, whereas that for paclitaxel was 12.8 months, and this difference is apparently significant (*p* = 0.033). PLD seems to be superior to paclitaxel in terms of PFS; however, the patients who received PLD were mostly diagnosed with AIDS-related KS (four of seven), and PLD is an approved agent against AIDS-related KS.

In an international multicenter analysis involving 55 patients with CKS treated with PLD as the first-line chemotherapy, the overall response rate was 71%, and the median PFS was 30 months. In that study, treatment was well-tolerated, and no toxicity-related death was reported [14]. In a retrospective study by Kreuter et al., PLD was compared with low-dose recombinant interferon (IFN) alfa-2a as treatment against advanced CKS; 11 of the 12 (92%) patients with CKS treated with PLD achieved a complete or major response, and one of the 6 (17%) patients treated with IFN alfa-2a achieved a major response (*p* < 0.05) [15]. In a large study involving 258 patients with AIDS-related KS, PLD was compared with the conventional DBV combination. In the PLD arm, the response ratio was significantly higher (46% vs. 25%) with less toxicity [16]. In another study, 241 patients with AIDS-related KS were divided into two arms. PLD was compared with the bleomycin and vincristine combination. The response rates were 59% and 23% for PLD and the bleomycin and vincristine combination, respectively [17]. In this study, the ORR and DCR were 57.1% and 100%, respectively, for the patients who received PLD in the first-line setting, and no cardiac or any side effects related to PLD were observed. The ORR in this study was lower than that reported in studies involving patients with CKS but was similar to that reported in studies involving patients with AIDS-related KS. A lower ORR was obtained in this study because most patients who received PLD had AIDS-related KS. Based on these data, PLD is apparently an effective and safe treatment option for patients with KS.

In this study, all six patients who received paclitaxel had non-AIDS-related KS. CR, PR, and SD were observed in two patients (33%), one patient (17%), and one patient (17%), respectively. In patients treated with paclitaxel, the ORR and DCR were 50% and 67%, respectively. In a retrospective study, 12 patients with non-AIDS-related, refractory, or life-threatening KS treated with taxanes (paclitaxel or docetaxel) were evaluated. In that retrospective study, the ORR was 100%, as all 12 patients had PR [18]. In another study, 17 patients CKS were treated with paclitaxel weekly. Due to their allergic reactions with paclitaxel, two patients were excluded from the study. Fourteen of the 15 patients had PR or CR, and the ORR was 93% [9]. Compared with the results of the aforementioned studies, the lower ORR obtained in this study can be attributed to our small sample size.

Visceral, lymph node, or bone involvement is normally associated with a poorer outcome, but when we compared PLD with paclitaxel in this subgroup, no statistical significance in terms of visceral, lymph node, or bone involvement was observed (*p* = 0.797) [19].

In this case series, the male-to-female ratio was 7:1; according to the European consensus-based interdisciplinary guideline for KS, the male-to-female ratios in Italy and Israel were 2:1 and 5:1, respectively [6]. In a retrospective study involving 18 patients with CKS recruited from three German centers, the male-to-female ratio was 5:1 [15]. Thus, the male-to-female ratio varies depending on the geographical area.

Furthermore, CKS is commonly seen between the fifth and eighth decades of life [20]. Consistent with the literature, the median age of the patients in this study was 61.07 years.

The limitations of our study include its small sample size, its retrospective nature, and the inclusion of patients with CKS and non-CKS. Besides, the toxicity data were mostly limited to laboratory findings due to the retrospective nature of this study.

## CONCLUSION

The real-world data showed that between the two most preferred chemotherapeutic agents, PLD seems better than paclitaxel in terms of PFS and response rates, and it demonstrated a good safety profile in patients with KS. There are no recommended globally approved chemotherapeutic regimens, particularly for CKS; thus, further large-scale prospective randomized studies with a longer follow-up period and more homogeneous patient populations are warranted to define the best therapeutic schedule.
